# Rapid determination of kappa-carrageenan using a biosensor from immobilized *Pseudomonas carrageenovora* cells

**DOI:** 10.1371/journal.pone.0214580

**Published:** 2019-04-16

**Authors:** Riyadh Abdulmalek Hassan, Lee Yook Heng, Asmat Ahmad, Ling Ling Tan

**Affiliations:** 1 School of Chemical Sciences and Food Technology, Faculty of Science and Technology, Universiti Kebangsaan Malaysia, UKM Bangi, Selangor Darul Ehsan, Malaysia; 2 Department of Chemistry, Faculty of Science, Ibb University, Ibb, Republic of Yemen; 3 Southeast Asia Disaster Prevention Research Initiative (SEADPRI-UKM), LESTARI, Universiti Kebangsaan Malaysia, UKM Bangi, Selangor Darul Ehsan, Malaysia; 4 School of Biosciences and Biotechnology, Faculty of Science and Technology, Universiti Kebangsaan Malaysia, Bangi, Selangor Darul Ehsan, Malaysia; The University of Akron, UNITED STATES

## Abstract

A potentiometric whole cell biosensor based on immobilized marine bacterium, *Pseudomonas carrageenovora* producing κ-carrageenase and glycosulfatase enzymes for specific and direct determination of κ-carrageenan, is described. The bacterial cells were immobilized on the self-plasticized hydrogen ion (H^+^)-selective acrylic membrane electrode surface to form a catalytic layer. Hydrogen ionophore I was incorporated in the poly(n-butyl acrylate) [poly(nBA)] as a pH ionophore. Catalytic decomposition of κ-carrageenan by the bienzymatic cascade reaction produced neoagarobiose, an inorganic sulfate ion and a proton. The latter was detectable by H^+^ ion transducer for indirect potentiometric quantification of κ-carrageenan concentration. The use of a disposable screen-printed Ag/AgCl electrode (SPE) provided no cleaning requirement and enabled κ-carrageenan detection to be carried out conveniently without cross contamination in a complex food sample. The SPE-based microbial biosensor response was found to be reproducible with high reproducibility and relative standard deviation (RSD) at 2.6% (n = 3). The whole cell biosensor demonstrated a broad dynamic linear response range to κ-carrageenan from 0.2–100 ppm in 20 mM phosphate buffer saline (PBS) at pH 7.5 with a detection limit at 0.05 ppm and a Nernstian sensitivity of 58.78±0.87 mV/decade (R^2^ = 0.995). The biosensor showed excellent selectivity towards κ-carrageenan compared to other types of carrageenans tested e.g. ι-carrageenan and λ-carrageenan. No pretreatment to the food sample was necessary when the developed whole cell biosensor was employed for direct assay of κ-carrageenan in dairy product.

## Introduction

Carrageenans are high molecular weight sulfated polysaccharides made up of repeating D-galactose residues and 3,6-anhydrogalactose, which are linked via alternating α-(1,3) and β-(1,4) linkages [1–2]. These high molecular weight polysaccharides are the major cell wall carbohydrate of various marine red algae species, and are common ingredient in many food items due to their thickening, gelling and stabilizing properties [3–4]. It is important to note that carrageenan is not digestible and has no nutritional value. It appears to be particularly destructive to the digestive system, triggering an immune response and causes inflammation, which can lead to gastrointestinal disease including ulcerative colitis, intestinal lesions and colon cancer. It is necessary to identify different types of carrageenans and detect them in foods because of the different toxicity levels of carrageenan to mammals with lambda carrageenan being the most toxic of all types of carrageenans to mammals [5]. There are three types of commercially available carrageenans such as kappa (κ)-, iota (ι)- and lambda (λ)-carrageenan, which are differentiated according to the number and position of sulfate ester group attached to the disaccharide units, and are used to manufacture a wide variety of commercial products e.g. milk products, processed meats, infant formula, toothpaste, cosmetics and pesticides [6]. Higher number of sulfate ester groups presence in the carrageenan molecule would result in lower solubility temperature and lower gel strength of the polysaccharide [6–8]. Typical sulfate levels in κ-, ι- and λ-carrageenan are 22.0% (w/w), 32.0% (w/w) and 38.0% (w/w), respectively [9–10], whilst the contents of carrageenans in different food products are ranging from 0.03% to 3.0% according to the purpose of their addition [11–12].

The most common challenge encountered with polysaccharide determination in foods by using high performance liquid chromatography (HPLC) and nuclear magnetic resonance (NMR) is that they cannot determine the position of sulfate group, and this hinders the accurate structural analysis of carrageenans. This makes differentiation between different types of carrageenans not possible [13–15]. A combination of chemical analysis approaches are usually needed involving gas liquid chromatography (GLC), spectrofluorometry, UV-Vis spectrophotometry and gel electrophoresis supplemented with HPLC or NMR in order to accurately determine the carrageenan type.

In general, biosensor is an analytical tool that built from immobilized biological molecules on a particular transducer, which transforms the biological recognition event into a measurable electrical response [16]. The biosensor provides a rapid, precise and accurate analytical tool for a wide variety of monitoring applications including environmental monitoring, bioprocess control, food quality control, military, medical diagnosis and agriculture [17–18]. Biorecognition elements normally take the form of a whole cell, enzyme, antibody, protein or nucleic acid. Based on recent studies, it is clear that enzymes have been widely used as bioreceptors in the fabrication of various biosensors [19–20]. While enzymes are sensitive to inhibitors, incorporation of enzyme molecules in biosensors may be limited because of the tedious, time-consuming and costly enzyme purification, the requirement for multiple enzymes to generate the measurable product, or the need for cofactor/coenzyme [21]. Whole cell biosensors, however, provide a quantification method because their components require no protein isolation and no purification steps as found in the cell component assay systems [20]. In addition, cells can simply adsorb on the transducer surface because of the membrane cell, which is composed of flagellar and fimbrial appendages, the outer membrane proteins, lipopolysaccharides and extracellular polysaccharides could give distinct physical and chemical interactions with different kinds of transducer surfaces [22–23]. There are various marine bacteria from the genus *Pseudomonas and Zobellia* secreting galactan endohydrolases, including those that are producing κ-carrageenases, ι-carrageenases and λ- carrageenases that are able to cleave randomly or at specific glycosidic bonds of carrageenan have been discovered [24]. Degradation of carrageenan by *P*. *carrageenovora* producing κ-carrageenase and λ- carrageenase [25–26], and *Alteromonas carrageenovora* producing ι-carrageenase [27], have also been reported for carrageenan bioassay. *P*. *carrageenovora* is generally found in connection with marine eukaryotes hosts, and their biological activities are important in the marine ecosystem. Many *Pseudomonas* species have been demonstrated to produce anti-bacterial products, agarases, toxins, bacteriolytic substances and other enzymes, which may facilitate the bacterial cells in their competition for nutrients, space and protection against predators in the marine environment [28].

**Fig 1:**
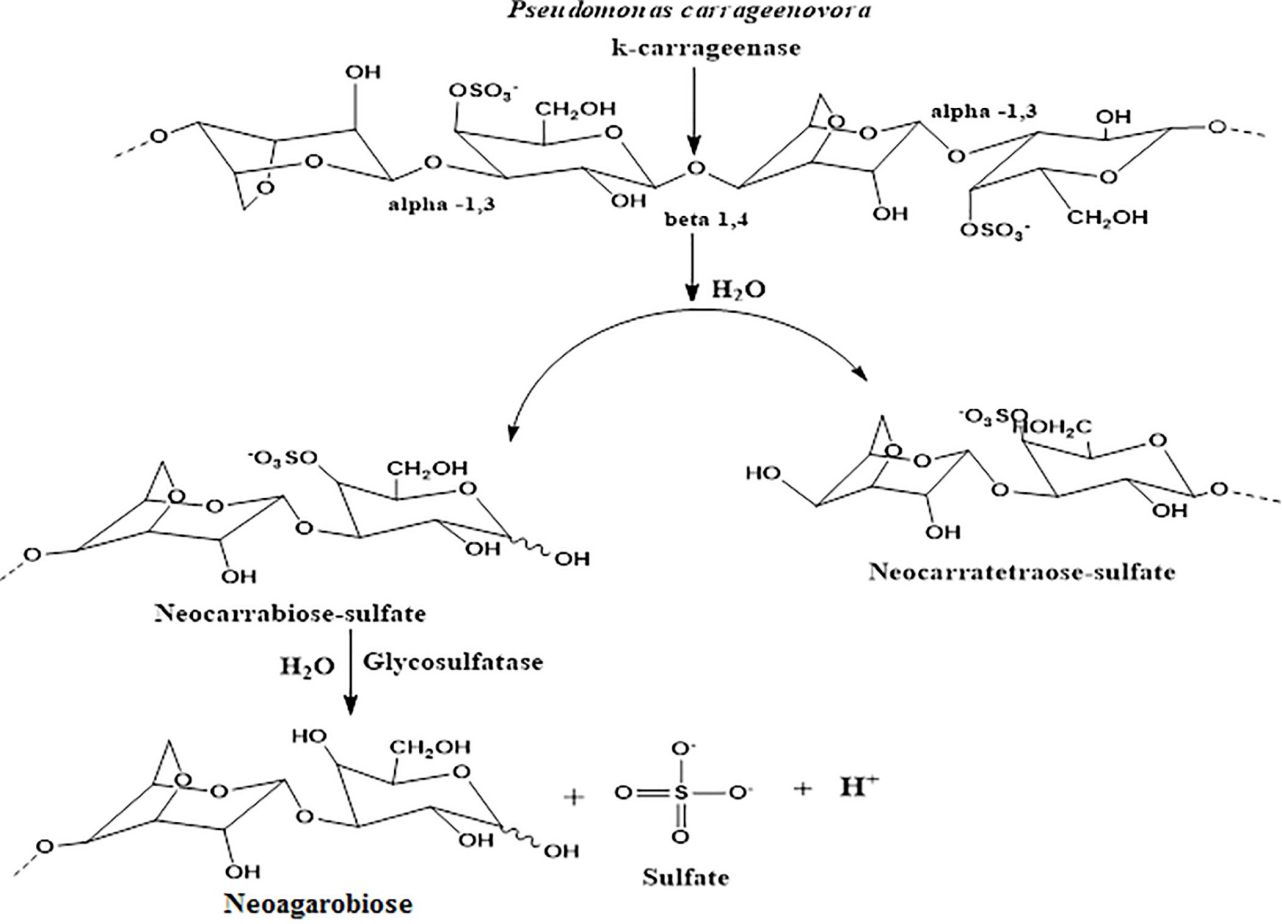
Schematic diagram of the bioenzymatic cascade reaction of κ-carrageenan by κ-carrageenase and glycosulfatase produced by the immobilized *P*. *carrageenovora* marine bacterial cells.

In this study, we present a simple potentiometric biosensor for direct detection of κ-carrageenan, being the most widely use carrageenan in the food industry as κ-carrageenan gel appears to be more firm than those obtained with ι- and λ-carrageenan. The proposed microbial potentiometric biosensor was constructed by using whole cells of *P*. *carrageenovora* through physical adhesion on the surface of a polyacrylate membrane. The membrane has the hydrogen ionophore I immobilized therein by gel entrapment technique on a screen-printed Ag/AgCl electrode (SPE). Immobilization of cells at the surface allows direct contact with the liquid phase containing the substrate, which promotes mass transfer kinetics without a diffusion barrier at the membrane surface [29]. In this potentiometric biosensor, it makes use of H^+^ ion-selective electrode in order to transduce the biological reaction into an electrical signal in the format of electromotive force (emf), which is the electrical potential difference generated between internal Ag/AgCl electrode and external reference electrode. The bioconversion of κ-carrageenan to neoagarobiose by the bienzymatic system consists of κ-carrageenase and glycosulfatase (Fig 1). The former enzyme catalyzes the hydrolytic cleavage of the internal β-(1,4) glycosidic bond of the κ-carrageenan to produce neocarrabiose sulfate [3-Ο-(3,6-anhydro-α-D-galactopyranosyl)-D-galactopyranose-4-O-sulfate] and neocarratetraose-sulfate [30–33]. At the same time, the latter enzyme catalyzes the enzymatic desulfation of neocarrabiose sulfate to neoagarobiose [3-O-(3,6-anhydro-α-L-galactopyranosyl)-D-galactopyranose], sulfate and hydrogen (H^+^) ions [25, 34–35]. An increasing H^+^ ion concentration detected by the H^+^ ion transducer would imply an increasing κ-carrageenan degradation rate by the immobilized marine bacterial cells at the SPE surface.

## Materials and methods

### Reagents and instrumentation

1000 mg L^-1^ stock solutions of κ- (Fluka), ι- and λ-carrageenan (Sigma) were prepared by dissolving 50 mg of the respective analytical grade carrageenans in 50 mL of deionized water. Further dilution of the carrageenan solutions were made with 20 mM phosphate buffer saline (PBS) at pH 7.0, which was prepared from the mixture of 20 mM Na_2_HPO_4_ (Hamburg Chemical) and NaH_2_PO_4_ (Fluka) solutions. Precursors for the plasticizer-free H^+^ ion-selective acrylic membrane was composed of n-butyl acrylate monomer (nBA, Merck), 2,2-dimethoxy-2-phenylacetophenone (DMPP, Fluka), 1,6-hexanediol diacrylate (HDDP, Sigma), sodium tetrakis[3,5-bis (trifluro-triethyl)phenyl]borate (NaTFPB, Fluka) and hydrogen ionophore I (Sigma). All chemicals used were of analytical reagent grade and used without further purification. All the solutions were prepared in Milli-Q deionized water (18 MOhm). A double junction Ag/AgCl reference electrode and the bacterial cells-modified SPE working electrode were connected to an ion meter (Orion) for potential (electromotive force) measurement. The bacterial cell concentration was measured with the UVmini-1240 Shimadzu Visible Spectrophotometer at the wavelength of 600 nm.

### Cultivation of bacteria

The culture medium was prepared according to the American Type Culture Collection (ATCC) procedure. In brief, 25 g NaCl, 5 g MgSO_4_.7H_2_O, 0.2 g CaCl_2_.2H_2_O, 0.1 g KCl, 2 g NaNO_3_, 2.5 g Casamino acids and 1.25 g κ-carrageenan were dissolved in 1 L deionized water followed by sterilization in an autoclave at 121°C for 20 min. 60 mL of sterilized 0.2 M Na_2_HPO_4_ and 10 mL of sterilized 0.3% FeSO_4_.7H_2_O were added aseptically into the growth medium, and adjusted to pH 7.2. *P*. *carrageenovora* IAM 12662 (ATCC), which were obtained as freeze-dried bacterial cells, were then cultured in 50 mL of the selective medium and grown for 48 h at 22°C in a 300 mL Erlenmeyer flask. After that, about 10% v/v bacterial culture was inoculated into the new culture medium and incubated for 24 h in an incubator shaker at 180 rpm. 600 mL of the culture medium was then centrifuged for 10 min at 3000 rpm and 4°C. The supernatant was discarded and the cell pellet was washed twice with 5 mM HEPES [4-(2-hydroxyethyl)-1-piperazineethanesulfonic acid] buffer via a quick vortex followed by centrifugation step. Finally, the bacterial cells were re-suspended in 20 mL of 5 mM HEPES buffer (pH 7.0) containing 0.1 M NaCl to maintain the bacterial membrane in a more fluid state, and kept at 4°C. The pour plat method was used as a reference method using LB broth agar serial dilutions of bacterial suspension with tenth strength prepared in HEPES buffer. 20 μL of each dilution was spread on the surface of culture media plate using glass. The number of bacterial colonies that formed on the surface of LB broth agar was calculated for each dilution, and the count lied between 30 and 300 colonies was selected to determine the number of bacterial colonies in bacterial colony-forming units per milliliter (CFU mL^-1^) unit. The bacterial number was calculated according to the following equation:

$$CFU\;mL^{‐1}=\frac{The\;number\;of\;bacterial\;colonies\times 1000}{20\times dilution\;factor}$$ (1)

The absorbance of the original suspsion was read against water at 600 nm, and was used as a reference reading for further bacterial concentration measurement.

### Whole cell biosensor preparation

**Fig 2:**
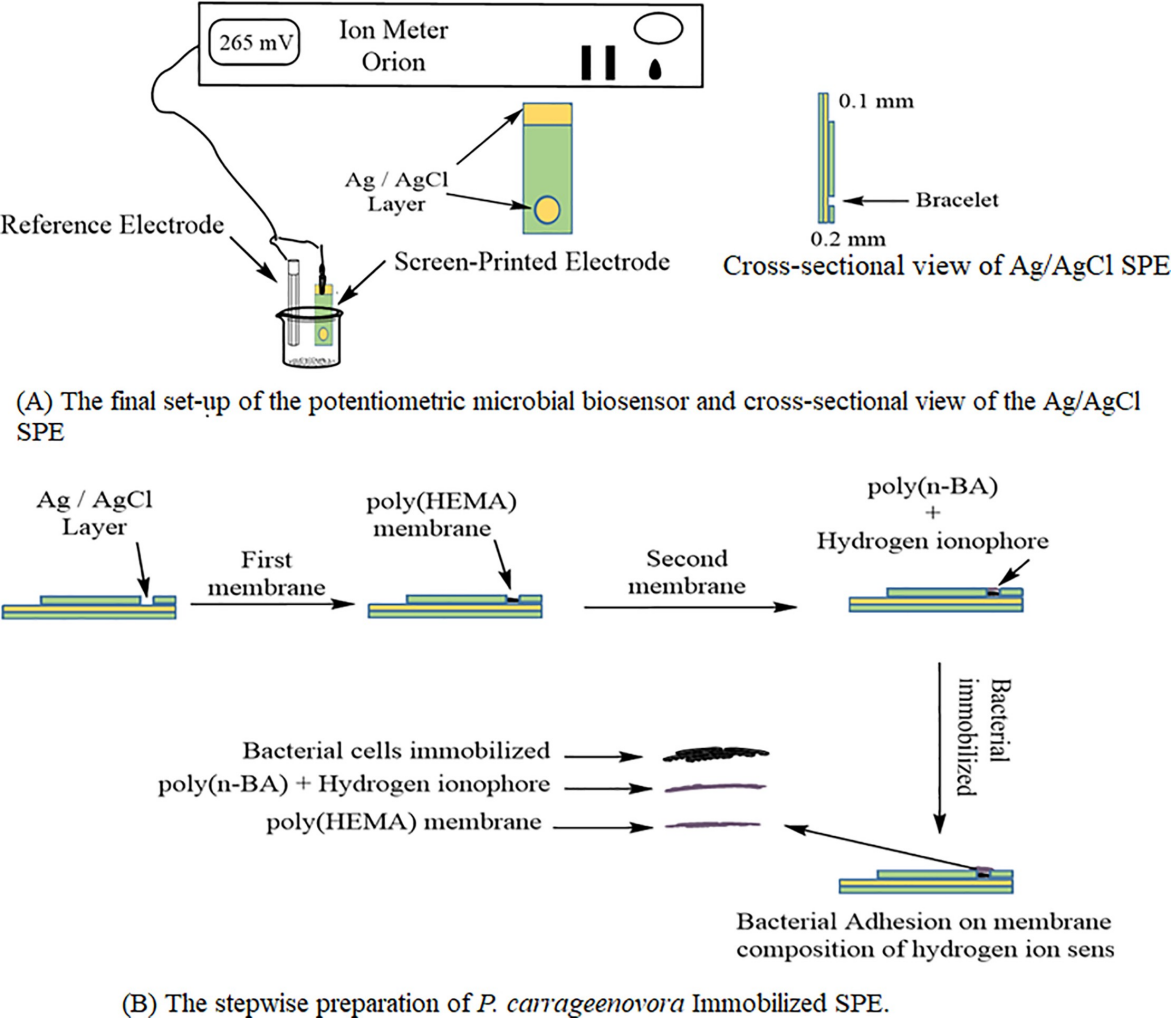
Potentiometric whole cell biosensor. (A) The final set-up of the potentiometric microbial biosensor and cross-sectional view of the Ag/AgCl SPE, and (B) the stepwise preparation of *P*. *carrageenovora* Immobilized SPE.

Screen-printed Ag/AgCl SPE was supplied by Scrint Technology (M) Sdn. Bhd (Fig 2). The SPE surface was first modified with an inner solution layer made of a photocurable poly(2-hydroxylethyl methacrylate) [poly(HEMA)] membrane. The poly(HEMA) membrane was prepared from 0.5 μL HEMA monomer containing 1.6 wt% DMPP photoinitiator followed by photocuring under UV radiation and nitrogen gas atmosphere in an UV-exposure unit (RS Ltd.) for 5 min. The hydrogel membrane was then hydrated with 0.01 M HCl solution for 15 min. Then, the membrane for H^+^ ion-selective sensor was deposited on top of the inner solution layer. The acrylic membrane was synthesized from 1 μL nBA monomer containing 1.2 wt% DMPP, 0.1 wt% HDDA cross-linker, 1.9 wt% hydrogen ionophore I and 43 mol% (relative to ionophore) NaTFPB, and photocured for another 5 min. The stacked membrane based-SPE was then washed several times with deionized water and air-dried before drop-coated with a 4 μL of bacterial suspension using a micropipette, and dried at 4°C for another 2 h.

### Optimization of microbial biosensor

The effect of cell loading was carried out by depositing 5 μL of different bacterial cell suspension concentrations on the surface of the H^+^ ion-selective poly(nBA) membrane on the SPE and dried at 4°C for 2 h. After that, the bacterial cells-modified electrode was immersed in 2 mL of 5 mM HEPES buffer (pH 7.5) containing 0.1 M NaCl until further use. Buffer capacity was optimized by varying the PBS concentration between 1 mM and 50 mM, whilst the buffer pH was optimized between pH 4 and pH 10 by using 20 mM PBS. Response time of the biosensor was assessed for 60 min towards the detection of 50 ppm κ-carrageenan in 20 mM phosphate buffer at pH 7.5. In order to determine the lifetime of the biosensor, 60 biosensors were prepared from the same batch, and stored in the refrigerator at 4°C. Three microbial biosensors were taken on the first day and their potentiometric responses were measured against the determination of 50 ppm κ-carrageenan in 20 mM phosphate buffer (pH 7.5), and the rest of the biosensors were evaluated intermittently over the experimental period of 60 days towards the same κ-carrageenan concentration at 50 ppm in 20 mM phosphate buffer at pH 7.5. Separate solution method (SSM) has been applied in the biosensor interference study by using κ-, ι- and λ-carrageenan as different substrates for the immobilized cells in the concentration range of 1–100 ppm. The developed potentiometric biosensor was then used for real sample study for recovery of a known amount of standard κ-carrageenan spiked into fresh milk. Optimum response of the biosensor was confirmed after statistical analyses of the obtained data using linear correlation statistics based on the slope of the response curve and t-tests of the highest slope values at p<0.001.

## Results and discussion

### Optimization of immobilized cell loading

**Fig 3:**
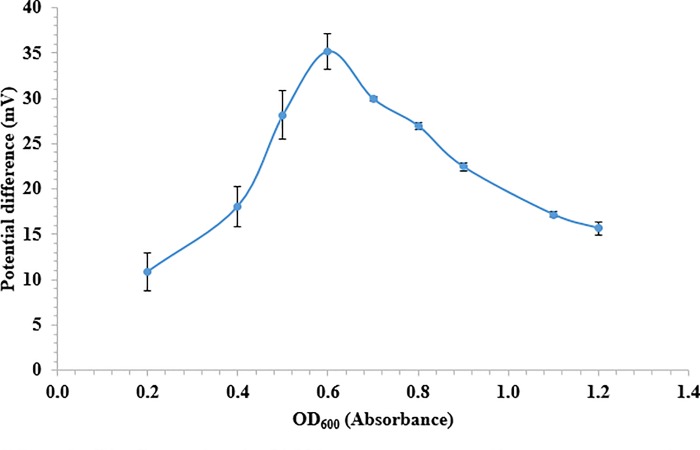
Effect of cell loading on the microbial biosensor response to 40 ppm κ-carrageenan in 10 mM PBS at pH 7.5 and 25°C (room temperature).

The immobilized *P*. *carrageenovora* concentration was studied to determine the optimum bacterial cell concentration for generating maximum potentiometric response. Changes in cell concentrations in suspension were measured by optical density (OD) at 600 nm. Fig 3 shows that the potentiometric signal increased with the increasing cell number (density) from 0.20 Abs to 0.60 Abs (0.1 unit of OD_600_ = 1×10^8^ cells/mL) immobilized at the H^+^ ion-selective electrode surface.

This can be explained by the fact that the enzymatic activity of the bacterial cell was increasing for the production of a higher amount of enzymes responsible for hydrolysis of κ-carrageenan followed by catalytic desulfation reaction to produce the proton. However, the potentiometric signal declined when the bacterial cell was loaded above 0.6 Abs because of the densely packed cells on the pH electrode forming a barrier for diffusion of κ-carrageenan to the immobilized cells. This limited the number of protons to reach to the transducer surface [35]. Optimizing the amount of bacterial cell immobilized on the H^+^ ion-selective acrylic membrane electrode surface was carried out in order to obtain maximum potentiometric response of the κ-carrageenan biosensor. Same result trending is observed in the previous study based on immobilized *Allivibrio fischeri* in alginate microspheres for fluorescence monitoring of heavy metal toxicity, whereby a decrement in the luminescence intensity of the biosensor was observed when the microbial cells loaded above 0.78 OD at 600 nm [36]. Therefore, the amount of cells was fixed at OD of 0.55–0.60 Abs in subsequent experiments. A similar response trend was also reported in some other constructions of microbial electrodes for the development of potentiometric and amperometric biosensors [37–39].

### pH effect and buffer capacity

The concentration of buffer solution can affect the activity of *P*. *carrageenovora*. The immobilized cell activity profile in different PBS concentrations at pH 7.0 is illustrated in Table 1.

**Table 1:** Effect of PBS concentration on the potentiometric response of the κ-carrageenan biosensor at pH 7 and room temperature (n = 4).

PBS concentration (mM)	Sensitivity (mV/decade)	R^2^	Linear range (ppm)
1	45.23±0.23	0.94	1–100
5	50.88±0.29	0.96	1–100
10	54.08±0.73	0.96	1–100
20	57.25±0.13	0.99	1–100
30	51.28±0.35	0.99	1–100
50	45.78±0.68	0.97	1–100

Higher buffer capacities at 20 mM PBS and above were found to give higher correlation coefficient values, whereby the regression lines fit the data well in a linear pattern. This is because a higher concentration of buffer can stabilize the pH change of reaction medium as the system involves proton-releasing enzymatic reaction. The near Nernstian pH response was attained with 20 mM PBS for κ-carrageenan detection between 1 ppm and 100 ppm, which is significantly higher (p<0.001) than other responses at various buffer capacities, hence this was served as the optimum buffer capacity for the whole cell biosensor.

The pH of the environment may also effect on the bacterial growth, thereby affecting cellular metabolism. Based on the results tabulated in Table 2, when the pH of the measurement cell is too high or too low, the sensitivity of the biosensor deviated substantially from the ideal Nernstian value (59 mV per decade) due to degradation of enzyme protein in both highly acidic and basic solutions. The sensitivities of the biosensor are not significantly different from pHs 7 to 8 statistically from each other. Thus, this is the optimum buffer pH for a good biosensor operation condition.

**Table 2:** Effect of buffer pH on the κ-carrageenan biosensor response in 20 mM PBS at room temperature (n = 3).

pH	Sensitivity (mV/decade)	R^2^	Linear range (ppm)
4.0	30.84±1.07	0.856	1–100
5.0	49.55±0.84	0.969	1–100
6.0	49.46±0.52	0.963	1–100
7.0	58.96±0.88	0.978	1–100
7.5	58.78±0.87	0.995	0.2–100
8.0	58.89±0.73	0.970	1–100
9.0	53.23±0.23	0.971	1–100
10.0	41.00±1.86	0.975	1–100

The biosensor exhibited good detection performance for κ-carrageenan between pH 7.0 and pH 8.0. The largest detection range of 0.2–100 ppm κ-carrageenan at pH 7.5 implies an optimum metabolic activity of the immobilized cells at this pH. Therefore, the PBS was maintained at pH 7.5 for further κ-carrageenan analysis with the potentiometric microbial biosensor.

### Response time and long term stability of the biosensor

**Fig 4:**
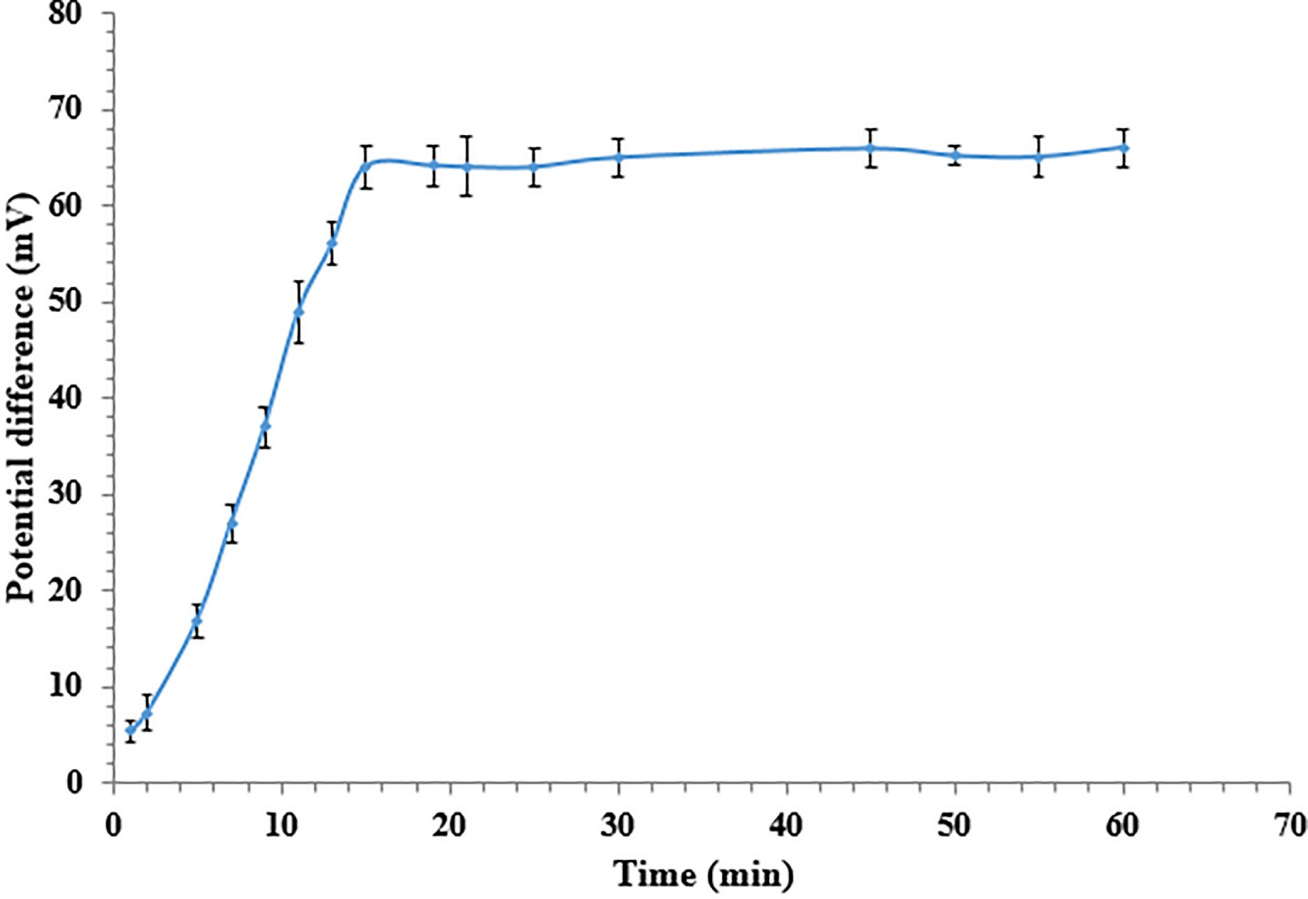
Response time of the biosensor towards 50 ppm κ-carrageenan in 20 mM phosphate buffer at pH 7.0 (n = 3).

The biosensor response time is controlled by the substrate diffusion through the membrane in addition to the cell or enzyme kinetics [40]. Fig 4 represents the response time profile of the potentiometric κ-carrageenan biosensor. Significant change in the biosensor response was observed in the first 10 min. The response time of the potentiometric whole cell biosensor towards 50 ppm κ-carrageenan in 20 mM phosphate buffer at pH 7.5 was determined at 15 min before the biosensor reached its steady state response, whereby a signal change of less than 4% per min after 15 min of reaction time was observed and remained rather constant thereafter.

**Fig 5:**
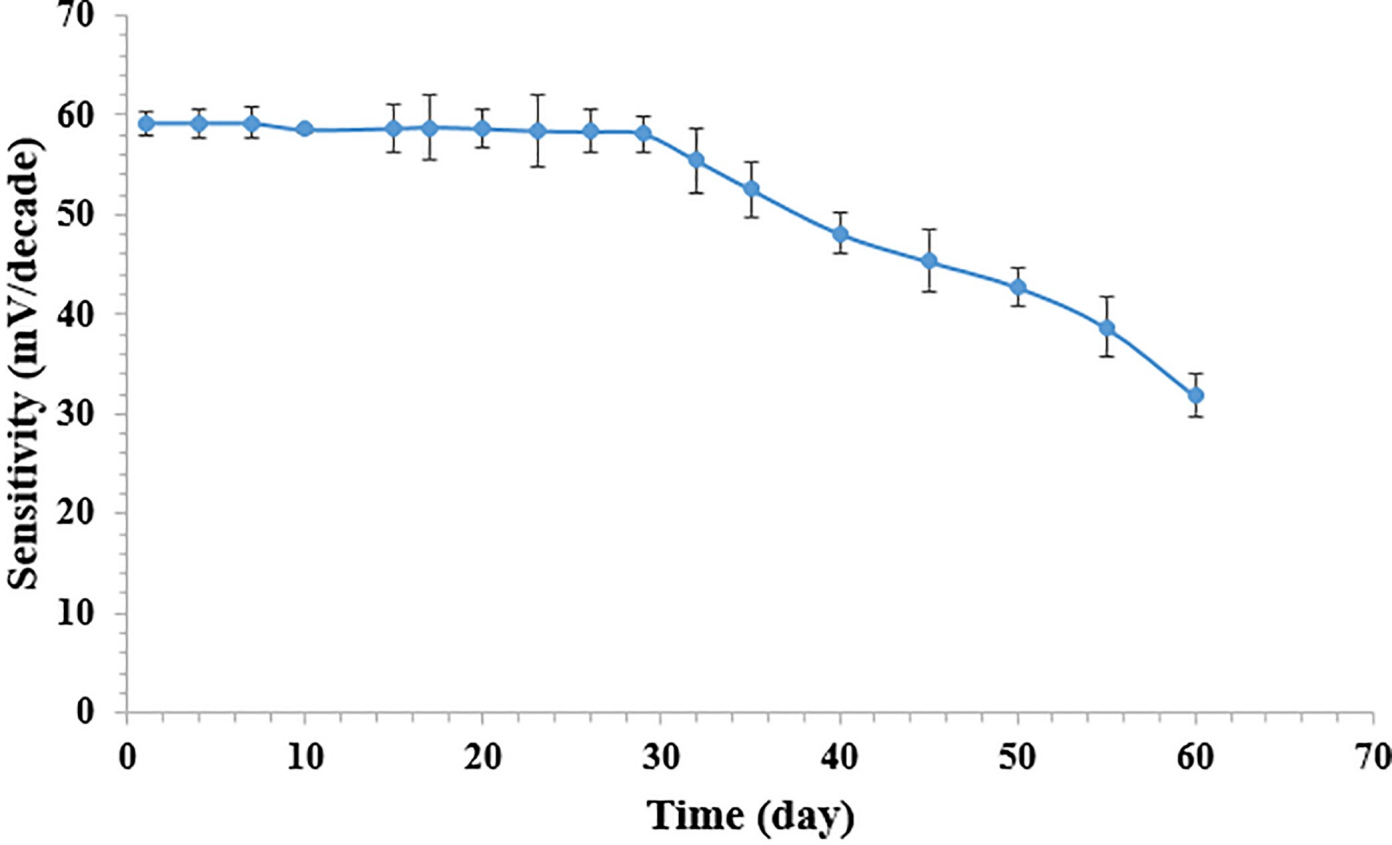
The changes of the potentiometric microbial biosensor response with time in 50 ppm κ-carrageenan at pH 7.5 (n = 3).

Bacterial cells are known for their instability with a limited life span. They are sensitive to changes in their environment and require a longer response time than its isolated components [41–42]. The stability of the immobilized cells determines the service life of the biosensor for practical applications. The operational life is also dependent on the immobilization method where a loss of cell activity could occur due to denaturation of cell [43]. In the present study, the potentiometric microbial biosensor stability was studied for 60 days. The changes in the potentiometric response of the biosensor over the experimental duration of 60 days towards a fixed amount of κ-carrageenan concentration at 50 ppm in 20 mM PBS (pH 7.5) is shown in Fig 5. The biosensor response was almost constant for the first 30 days and gradually decreased thereafter until the 45^th^ day of the experimental period with only 65% of the initial response remaining. Thus, the biosensor operational period was approximately 20 days.

### Biosensor selectivity

**Fig 6:**
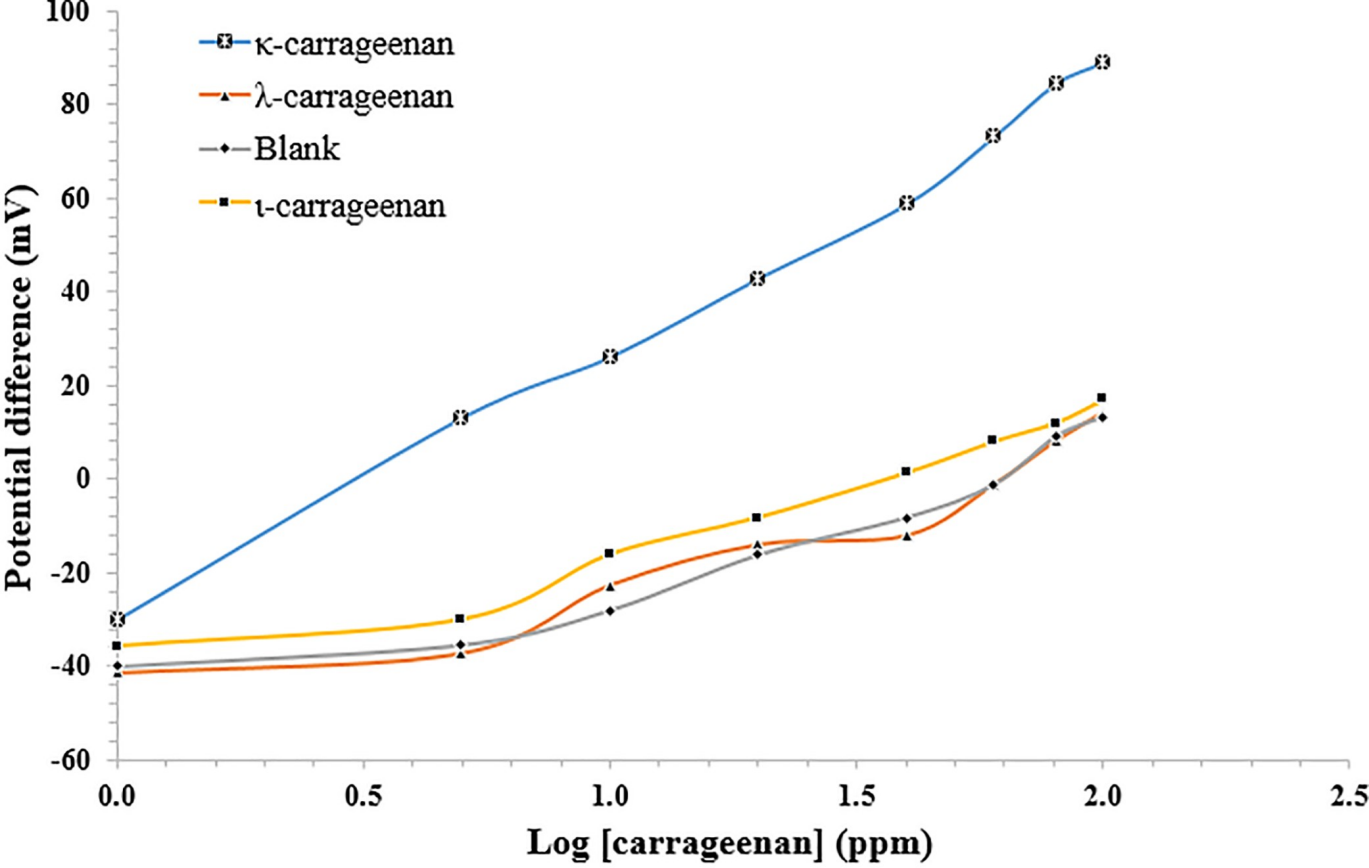
The kinetic curves of the potentiometric biosensor towards 1–100 ppm κ-, ι- and λ-carrageenan by using SSM method.

The selectivity and specificity of a biosensor depends on the biological recognition system that is connected to a transducer [44]. Enzymes are known for their high selectivity to their specific analytes. The selectivity of the *P*. *carrageenovora*–based biosensor towards κ-carrageenan was conducted based on SSM method by IUPAC, whereby the potentiometric responses of the microbial biosensor towards κ-carrageenan, ι-carrageenan and λ-carrageenan were evaluated separately in the concentration range of 1–100 ppm. These carrageenans were chosen because they are common gelling agents applied in modern food technology. Fig 6 and Table 3 shows the carrageenan effect on the electrochemical biosensor response.

**Table 3:** The selectivity of the potentiometric biosensor towards κ-, ι- and λ-carrageenans.

Analyte	Sensitivity (mV/decade)	R^2^	Linear range (ppm)
κ-carrageenan	58.77	0.99	1–100
ι-carrageenan	27.80	0.95	1–100
λ-carrageenan	27.67	0.90	1–100
Blank	27.72	0.90	1–100

The biosensor exhibited a significantly higher response towards κ-carrageenan when compared with other types of carrageenan (p<0.001) and negligible responses were obtained from both ɩ- and λ-carrageenan over the concentration range of 1–100 ppm, which suggests good selectivity of the potentiometric whole cell biosensor towards κ-carrageenan. The SPE-based biosensor gave satisfactory relative standard deviation (RSD) at 2.6% for three consecutive κ-carrageenan measurements with three different biosensor electrodes indicating high reproducibility of the sensing elements for measurement of κ-carrageenan.

### Recovery study

The developed potentiometric microbial biosensor has been applied for the determination of standard κ-carrageenan added into a fresh milk sample. Recoveries of κ-carrageenan between 94.0% and 99.8% for 4–30 ppm spiked standard κ-carrageenan indicates that the biosensor can be used for accurate and reliable determination of κ-carrageenan in dairy milk sample (Table 4).

**Table 4:** Recovery of κ-carrageenan in milk sample with the potentiometric whole cell biosensor.

Spiked κ-carrageenan concentration (ppm)	κ-carrageenan concentration determined by biosensor (ppm)	% Recovery
4	3.76 ± 0.04	94.0
8	7.92 ± 0.07	99.0
15	14.87± 0.19	99.1
30	29.95 ± 0.17	99.8

The proposed potentiometric whole cell biosensor based on marine bacterial cells could sensitively detect the concentration of κ-carrageenan at low levels via degradation of κ-carrageenan compared to the previously reported electrochemical potentiometric titration techniques using immobilized dinonylnaphthalene sulfonate (DNNS) [45] or tridodecylammonium chloride (TDMAC) ionophore [11] in polymeric membranes e.g. poly(vinyl chloride) and polyurethane, and protamine or poly-L-arginine used as the titrant [11, 45] (Table 5). The fabricated microbial biosensor exhibited a broader dynamic concentration detection range for carrageenan, i.e. approximately 5 orders of magnitude wider than the previously reported potentiometric titration methods [11, 45] and chronopotentiometric stripping method [46] for quantitation of κ-carrageenan. This was attributed to the surface functionalization of acrylic membrane with microbial cells such that they can directly access to the carrageenan molecule in the solution, hence tends to be much more sensitive towards degradation of carrageenan. Entrapment method of immobilization within polymeric membrane, on the other hand, is based on the localization of sensing element within a polymer matrix, which can render the resulting sensing phase sustains lower sensitivity, poor selectivity and limited stability. The reagent molecule embedded in the polymer matrix could restrict the diffusion of substrate to the active site of immobilized receptor, thereby hampers the internal diffusion of large carrageenan molecules within the substrate matrix. The developed potentiometric microbial biosensor has several advantages over previously reported electrochemical methods in being more sensitive, facile, stable and feasible to be employed for direct carrageenan analysis in complex food samples especially in confectionery and dairy products without sample pre-treatment.

**Table 5:** Comparison between the developed potentiometric κ-carrageenan whole cell biosensor performance and the previously reported potentiometric biosensor for carrageenan.

Parameter	Present study	[11]	[45]	[46]
linear range (ppm)	0.2–100	1.9–19.9	2–20	18–90
Sensitivity (mV/decade)	58.78±0.87	18.8	-	-
Response time (min)	15	5	-	5
Limit of detection (ppm)	0.05	0.70	0.2	18
Lifetime (day)	30	-	-	-

## Conclusions

The whole cell biosensor based on *P*. *carrageenovora* bacterium is advantageous for simpler and faster detection of κ-carrageenan compared to other previously reported methods that involve a combination of laboratory-scale analytical instruments. Furthermore, the biosensor based on disposable SPE is low-cost and provides portable version of test device for on-site routine analysis to be carried out in a convenient manner. The electrochemical potentiometric detection method shortens the analysis time to a few minutes as compared to the hours-long spectrophotometric method. In addition, it is also useful for assay of κ-carrageenan contents in highly coloured and cloudy samples, being not susceptible to dyeing interference.
